# Supplementary material for: PBP4 Is Likely Involved in Cell Division of the Longitudinally Dividing Bacterium Candidatus Thiosymbion Oneisti
Source: Antibiotics (Basel). 2021 Mar 9;10(3):274. doi: 10.3390/antibiotics10030274 (PMC7999549; doi:10.3390/antibiotics10030274)
Supplement: Supplementary file 1 [file antibiotics-10-00274-s001.pdf]

antibiotics

MDPI

Article

PBP4 is Likely Involved in Cell Division of the Longitudinally Dividing Bacterium *Candidatus* Thiosymbion Oneisti

Jinglan Wang [1], Laura Alvarez [2], Silvia Bulgheresi [3], Felipe Cava [2] and Tanneke den Blaauwen [1,*]

1 Bacterial Cell Biology & Physiology, Swammerdam Institute for Life Sciences, Faculty of Science, University of Amsterdam; Science Park 904, Amsterdam 1098 XH, The Netherlands; J.Wang3@uva.nl
2 Department of Molecular Biology, Umeå University, Umeå SE-901 87, Sweden; laura.alvarez@umu.se (L.A.); felipe.cava@umu.se (F.C.)
3 Environmental Cell Biology, University of Vienna, Althanstrasse 14 (UZA I), Vienna 1090, Austria; bulghes3@univie.ac.at
* Correspondence: T.denblaauwen@uva.nl

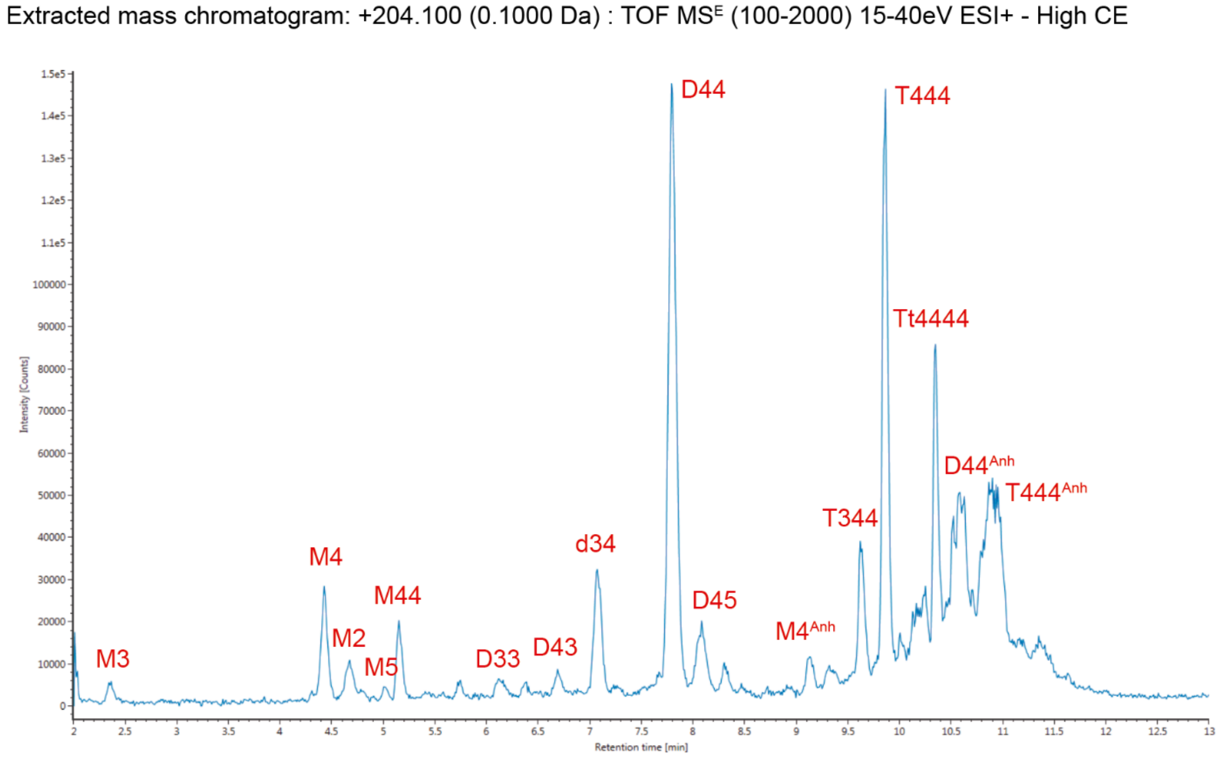

Figure S1. Muropeptide profile of *Ca.* T. oneisti obtained by LC-MS. Masses of indicated peaks are shown in supplementary table S3.

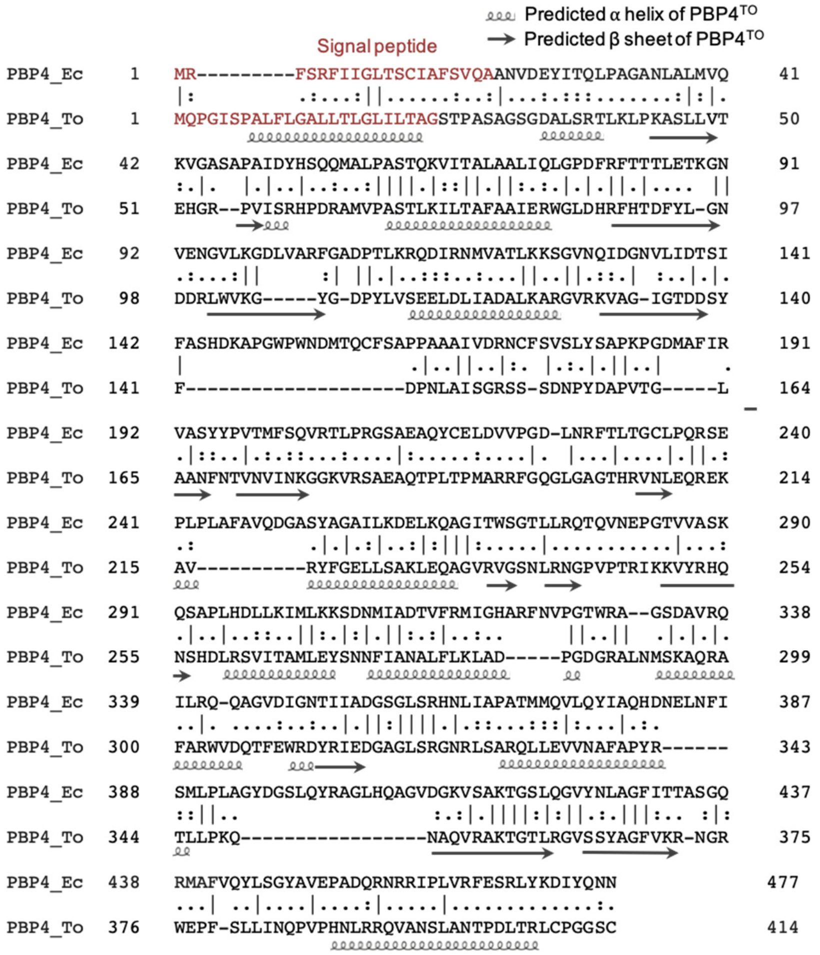

Figure S2. Alignment of PBP4EC and PBP4TO.

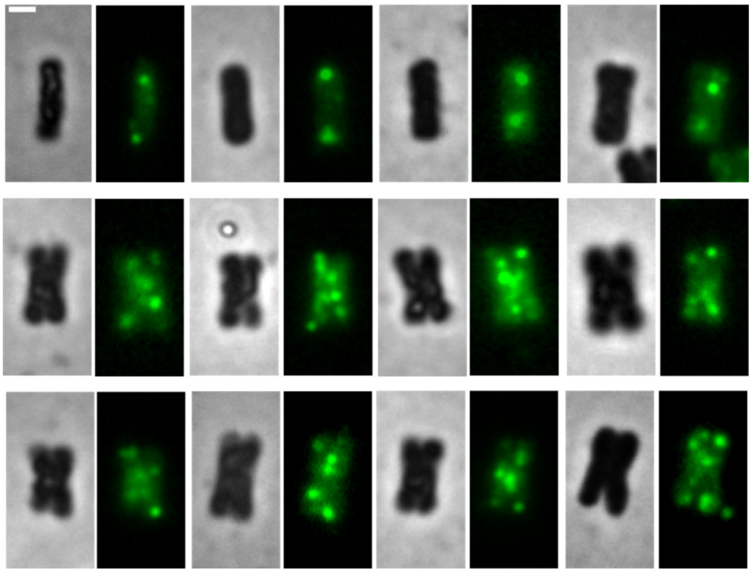

Figure S3. PBP4 localization in *Ca.* T. oneisti. Phase contrast and fluorescence images of representative *Ca.* T. oneisti cells of PBP4 immunolabeling.

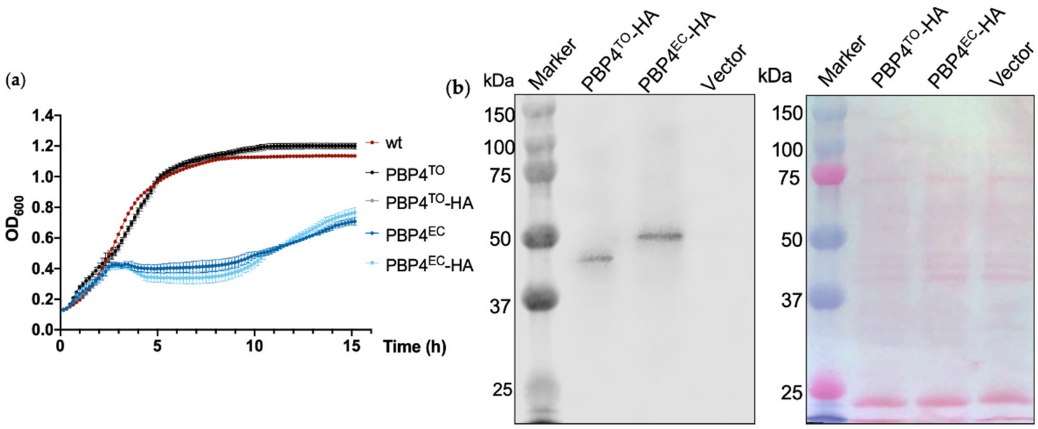

Figure S4. PBP4TO/EC protein expression level. (a) Growth curves of wild type E. coli with PBP4EC/TO from plasmid pXL133/pJW05 or PBP4EC/TO-HA overexpression from plasmid pJW08/pJW09 in-duced with 200 μM IPTG in TY medium at 28 °C. The values are the mean ± SD from three re-peats. (b) Western blot image and corresponding Ponceau S stain shows the expression level of PBP4TO/EC in wild type E. coli. induced with 50 μM IPTG for 4h cultured in TY medium at 28 °C.

Table S1. Muropeptides of *Ca.* T. oneisti analyzed by LC-MS.

No.	Name	Structure	RT (min)	Neutral Mass (Da)		Difference	RA (%)
				Theoretical	Observed		
**1**	**M3**	**GlcNAc-MurNAc-L-Ala-D-Glu-DAP**	**2.36**	**870.3706**	870.3665	0.0041	1.57
2	M4	GlcNAc-MurNAc-L-Ala-D-Glu-DAP-D-Ala	4.43	941.4077	941.4066	0.0011	24.77
3	M2	GlcNAc-MurNAc-L-Ala-D-Glu	4.68	698.2858	698.2856	0.0002	8.33
4	M5	GlcNAc-MurNAc-L-Ala-D-Glu-DAP-D-Ala-D-Ala	5.00	1012.4448	1012.4487	-0.0039	1.32
5	M44	M4-M4 (cross-linked at D-Ala-DAP) with loss of a disaccharide	5.15	1384.6093	1384.6172	-0.0079	7.68
6	D33	M3-M3 (cross-linked at DAP-DAP)	6.12	1722.7306	1722.7372	-0.0066	0.35
7	D43	M4-M3 (cross-linked at D-Ala-DAP)	6.69	1793.7677	1793.7773	-0.0096	1.09
8	D34	M3-M4 (cross-linked at DAP-DAP)	7.07	1793.7677	1793.7773	-0.0096	5.77
9	D44	M4-M4 (cross-linked at D-Ala-DAP)	7.79	1864.8048	1864.8019	0.0029	29.72
10	D45	M4-M5 (cross-linked at D-Ala-DAP)	8.08	1935.8420	1935.8412	0.0007	0.04

11	M4^{Anh}	GlcNAc-(1-6anhydro)MurNAc-L-Ala-D-Glu-DAP-D-Ala	9.14	921.3815	921.3794	0.0021	1.48
12	T344	M3-M4-M4 (crosslinked at DAP-DAP and D-Ala-DAP)	9.62	2717.1649	2717.0087	0.1562	0.10
13	T444	M4-M4-M4 (crosslinked at D-Ala-DAP)	9.87	2788.2020	2788.2043	-0.0023	10.29
14	Tt4444	M4-M4-M4-M4 (crosslinked at D-Ala-DAP)	10.35	3711.5991	3711.3680	0.2311	1.01
15	D44^{Anh}	M4-M4A (cross-linked at D-Ala-DAP)	10.62	1844.7786	1844.7692	0.0094	6.32
16	T444^{Anh}	M4-M4-M4A (crosslinked at D-Ala-DAP)	10.90	2768.1758	2768.1799	-0.0041	0.16

RA = Relative molar abundance.

Table S2. E. coli strains and plasmids used in this study.

***E. coli* strain**	**Relevant Genotype**	**Reference**
LMC500	MC4100 *lysA*	[1]
ΔPBP4	BW25113Δ*dacB*	[2]
SF100	KS272 Δ*ompT*	[3]
D456	*his supF ΔdacA*::Km *dacB*::Spc *ΔdacC1*	[4]
**Plasmids**	**Property**	**Reference or source**
pJW05	pTrc 99A down expressing PBP4^{TO}, p15A ori, CmR	This work
pJW06	pTrc 99A expressing PBP4^{TO}-8His, ColE1 ori, CmR	This work
pJW07	pTrc 99A expressing PBP4^{TO} S69A-8His, ColE1 ori, CmR	This work
pJW08	pTrc 99A down expressing PBP4^{TO}-HA, p15A ori, CmR	This work
pXL133	pTrc 99A down expressing PBP4^{EC}, p15A ori, CmR	[5]
pJW09	pTrc 99A down expressing PBP4^{EC}-HA, p15A ori, CmR	This work

Table S3. Primers used in this study.

**Primer**	**Sequence 5′-3′**	**use**
priJW84	ATGCAACCCGGAATATCCCCAGC	pJW06 construction
priJW85	TCAACAACTCCCGCCCGGGCACA	pJW06 construction
priJW86	TGGGGATATTCCGGGTTGCATGGCTT-GTCATGTCATCG	pJW06 construction
priJW87	TGCCCGGGCGGGAGTTGTT-GATGGTCGACCTGCAGGCATGC	pJW06 construction
priJW88	TCACACAGGAAACAGACCATGTTT-GAACCAATGGAACT	pJW05 construction
priJW89	GTTCGGGCCCAA-GCTCATTATTAATCAGCTTGCTTACGC	pJW05 construction

priJW105	TTCCGGCGGCAACGCTGAA-GATCCTGACCGCCTTCG	pJW07 construction
priJW106	ATCTTCAGCGTTGCCGCCGGAAC-CATGGCCCGGTCC	pJW07 construction
priJW111	GGTGGTTCTTACCCATACGATGTTCCA GATTACGCTTGAA AGCTTGGGCCCGAACA	pJW08 and pJW09 construction
priJW112	ATCGTATGGGTAAGAACCAC-CAGAACCACCACAACTCC CGCCCGGGCACA	pJW08 construction
priJW114	ATCGTATGGGTAAGAACCAC-CAGAACCACCATTGTTCTG ATAAATATCTTTATACAA	pJW09 construction

Reference

1. Taschner, P. E.; Verest, J. G.; Woldringh, C. L. Genetic and morphological characterization of ftsB and nrdB mutants of *Escherichia coli*. *J. Bacteriol* **1987**, *169*, 19–25.
2. Baba, T.; Ara, T.; Hasegawa, M.; Takai, Y.; Okumura, Y.; Baba, M.; Datsenko, K. A.; Tomita, M.; Wanner, B. L.; Mori, H. Construction of *Escherichia coli* K-12 in-frame, single-gene knockout mutants: the Keio collection. *Mol Syst Biol* **2006**, *2*, 2006–2008.
3. Baneyx, F.; Georgiou, G. In vivo degradation of secreted fusion proteins by the *Escherichia coli* outer membrane protease OmpT. *J. Bacteriol* **1990**, *172*, 491–494.
4. Edwards, D. H.; Donachie, W. D. Construction of a Triple Deletion of Penicillin-Binding Proteins 4, 5, and 6 in *Escherichia coli*. In *Bacterial Growth and Lysis*; Metabolism and Structure of the Bacterial Sacculus; Springer, Boston, MA: Boston, MA, 1993; Vol. 172, pp. 369–374.
5. Verheul, J.; Lodge, A.; Yau, H.; Liu, X.; Solovyova, A. S.; Typas, A.; Banzhaf, M.; Vollmer, W.; Blaauwen, den, T. Midcell localization of PBP4 of *Escherichia coli* is essential for the timing of divisome assembly. *bioRxiv* **2020**, *32*, 149–54.
